# Case report: Corticosteroids as an adjunct treatment for the management of liver abscess in Papillon–Lefèvre syndrome: A report on two cases

**DOI:** 10.3389/fped.2022.953033

**Published:** 2022-09-20

**Authors:** Suprit Basu, Reva Tyagi, Ankur Kumar Jindal, Apurva Medha, Aaqib Zaffar Banday, Alisha Babbar, Apoorva Sharma, Rahul Mahajan, Pandiarajan Vignesh, Amit Rawat

**Affiliations:** ^1^Pediatric Allergy Immunology Unit, Department of Pediatrics, Postgraduate Institute of Medical Education and Research, Chandigarh, India; ^2^Department of Dermatology, Venereology and Leprology, Postgraduate Institute of Medical Education and Research, Chandigarh, India

**Keywords:** corticosteroids, Papillon–Lèfevre syndrome, liver abscess, surgical management, *Staphylococcus aureus*

## Abstract

Papillon–Lefèvre syndrome (PLS) is a rare autosomal recessive disorder characterized clinically by palmoplantar keratoderma, periodontitis, and recurrent pyogenic infections. Liver abscess is rarely reported in patients. The use of corticosteroids for the treatment of liver abscess akin to chronic granulomatous disease (CGD) has not been reported previously. Here, we report 2 cases of liver abscess in PLS that responded to corticosteroids.

## Introduction

Papillon–Lefèvre syndrome (PLS) is a rare autosomal recessive disorder characterized clinically by palmoplantar keratoderma and periodontitis affecting both primary and permanent dentitions (1). Patients with PLS also have immune abnormalities, especially defects in neutrophils, and are predisposed to develop recurrent pyogenic infections. Liver abscess is increasingly being reported in patients with PLS (2). Majority of patients have been reported to respond to antimicrobials and surgical drainage. However, there are no reports on the use of corticosteroids for liver abscess in patients with PLS. Corticosteroids have been reported to be useful for the management of liver abscess in patients with chronic granulomatous disease (CGD). This suggests the presence of a hyperinflammatory response around the abscess. We report 2 patients with PLS who liver abscess was successfully resolved after administration of corticosteroids.

## Case report

### Case 1

A 5-year-old boy, born out of non-consanguineous marriage, presented with high-grade fever and right-sided upper abdominal pain. He developed liver abscess at the age of 3 years, and the pus culture grew methicillin-resistant *Staphylococcus aureus* (MRSA). He was admitted and received intravenous antibiotics for 14 days in a peripheral center.

Now at 5 years of age, he had hepatomegaly and palmoplantar keratoderma. There was no dental abnormality. Laboratory investigations showed anemia, neutrophilic leucocytosis, high erythrocyte sedimentation rate (ESR), elevated C- reactive protein (CRP), and high serum immunoglobulins (Table 1). Ultrasonography (USG) of the abdomen revealed a 10 × 8 cm abscess involving segments IV and VIII of the liver. Pigtail drainage of the abscess was conducted, and the pus grew methicillin-sensitive *Staphylococcus aureus* (MSSA). He was initiated on cloxacillin and vancomycin. However, he continued to have fever even after 14 days of antibiotics. Acute phase reactants were also elevated. Open surgical drainage of the abscess was planned. However, the intraoperative findings revealed no drainable collection, and only organized calcified pus was noticed. The pus culture was sterile, and the biopsy revealed a fibrocollagenous tissue. In view of the persistent fever, he was initiated on oral prednisolone (1 mg/kg/day). There was prompt defervescence and normalization of acute phase reactants. The size of liver abscess was reduced within 7 days of the initiation of prednisolone. Prednisolone was continued for 4 weeks and tapered off over the next 2 weeks. The follow-up ultrasound showed no liver abscess. The targeted next-generation sequencing (*invitae* primary immunodeficiency gene panel) showed a novel pathogenic homozygous complex rearrangement in the *CTSC* gene (resulting in the deletion of exon 1, which includes the initiator codon, and exon 3).

**TABLE 1:** Laboratory investigations.

Parameters	Case 1	Case 2
Hemoglobin (g/L)	92	89
White blood cell count (× 10^9^/L)	23.3	26.3
Neutrophils/lymphocytes/ monocytes/eosinophils (%)	81/16/2/1	70/20/6/4
Platelets (×10^9^/L)	200	170
Erythrocyte sedimentation rate (mm in 1st h) (Normal < 20)	88	69
C reactive protein (mg/L) (normal < 6)	119	96
Nitroblue tetrazolium dye reduction test Dihydrorhodamine assay	Normal Normal	Normal Normal
% CD3 + T cell (N-56–75%;1.4–3.7/L) % CD19 + B cell (N-14–33%;0.39–12.4/L) % CD56 + NKcell (N-4–17%; 0.13–0.72/L)	77.42% (2.87/L) 14.74% (0.55/L) 7.81% (0.291/L)	68% (3.576/L) 17.7% (0.931/L) 6.9% (0.362/L)
Immunoglobulin G (g/L) (Normal 4.9–16.1) Immunoglobulin M (g/L) (Normal 0.5–2.0) Immunoglobulin A (g/L) (Normal 0.5–2.4)	22.2 3.27 4	20 4 3
Immunoglobulin E (Normal < 100 U/L)	66.4	64

He is on follow-up for 18 months, is taking cotrimoxazole prophylaxis, and is doing well.

### Case 2

A 6-year-old girl, born out of non-consanguineous marriage, presented with high-grade fever and right upper quadrant abdominal pain. At 4.5 years, she had liver abscess (3.4 cm × 3.5 cm × 3.1 cm) in segment III. The lesion was extending into the sub-capsule. She was managed in another healthcare facility, and a pig-tail catheter was inserted for drainage of the pus. The pus culture grew MRSA. She was treated with intravenous antimicrobials for 6 weeks. Five months later, she had recurrence of the liver abscess (pus grew MRSA) that was also managed in another healthcare facility.

**FIGURE 1:**
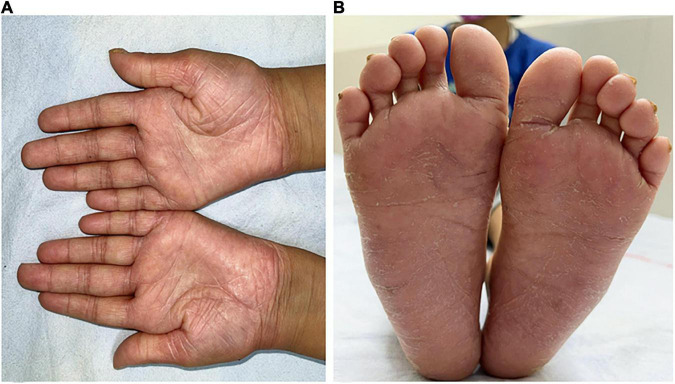
Diffuse erythematous hyperkeratotic scaling and fissuring in the **(A)** palm and **(B)** sole **(B)** of case 2.

**FIGURE 2:**
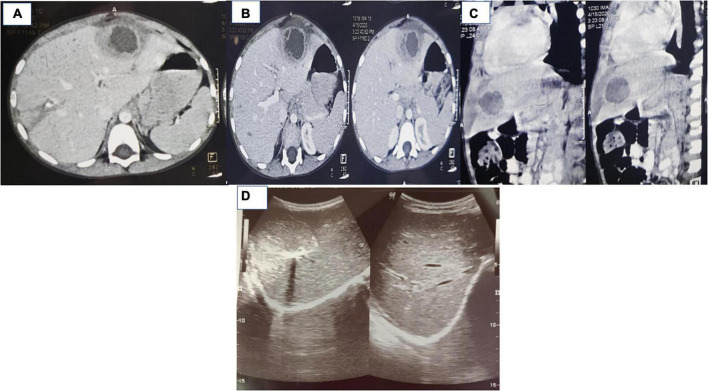
**(A–C)** Computed tomography of case 2 showing a hypoattenuating focal lesion with thick/shaggy walls with perilesional edema suggestive of abscess in the liver, arrow marks. **(D)** Ultrasound images of case 2 showing resolution of abscess following initiation of corticosteroids (after 6 weeks of initiation of therapy).

On examination, she had thickened and keratotic skin over palms and soles, dental caries, and hepatomegaly (Figure 1). Laboratory investigations showed anemia, neutrophilic leucocytosis, and high ESR, CRP, and serum immunoglobulins (Table 1). Before presenting to us, she had undergone computed tomography (CT) of the abdomen that showed a heterogeneous area measuring 3.3 cm × 2.6 cm × 2.3 cm in size seen in segment III of the liver (Figures 2A–C).

The USG abdomen carried out in our institute showed multiple organized abscesses in the right lobe of the liver in segment VII/VIII (largest: 6.9 cm × 5.1 cm) and another abscess in segment VI (4.1 cm × 4.5 cm). The orthopantogram was normal. USG-guided fine-needle aspiration cytology for organized liver abscess was performed. The pus culture was sterile. She was empirically initiated on cloxacillin and vancomycin. For keratoderma, emollients, coconut oil, topical steroids, and calcitriol ointment were advised. However, she continued to be febrile after 2 weeks, and there was no decrease in abscess size. She was initiated on oral prednisolone (1 mg/kg/day) and continued on intravenous antimicrobials. She showed prompt recovery and became afebrile within 24 h. The USG abdomen showed a marked reduction in the size of the liver abscess (2.1 cm × 2.3 cm, 7 days after initiation of prednisolone). Prednisolone was continued for 6 weeks and tapered off in another 6 weeks. The whole exome sequencing showed a homozygous mutation in the *CTSC* gene resulting in deletion of exons 1–17.

The follow-up USG at 6 weeks showed 1 × 1 cm residual liver abscess (Figure 2D). She is being continued on cotrimoxazole prophylaxis (5 mg/kg/day of trimethoprim component) and has no recurrence of liver abscess in 2 months follow-up.

Herein, we report 2 patients with PLS with liver abscess that showed brisk improvement following the administration of corticosteroids.

## Discussion

PLS is a rare autosomal recessive disorder characterized clinically by palmoplantar keratoderma, periodontitis, and recurrent pyogenic infections (1). PLS is caused by a homozygous or compound heterozygous mutation in the *cathepsin C* gene (CTSC). CTSC has a role in the activation of azurophilic granules in neutrophils and granzyme secretion in cytotoxic T lymphocytes. Patients with PLS lack active serine proteases in neutrophils and cytotoxic T lymphocytes and have decreased neutrophil extracellular trap formation in response to reactive oxygen species (2). This predisposes them to develop recurrent infections.

Progressive periodontitis leading to premature loss of teeth, gingival inflammation, and rapid destruction of periodontium is the hallmark manifestation (3). Loss of teeth usually occurs during the teenage years, but the third molar may be spared (4). Periodontitis and loss of teeth were, however, not observed in both patients. Strict oral hygiene and careful management of infections may be required to prevent the development of this complication (4).

Palmoplantar keratoderma is another hallmark feature and usually develops by the age of 4. Hyperhidrosis may also be seen. Skin histopathology may reveal non-specific hyperkeratosis, focal parakeratosis, dilated tortuous capillaries in dermal papillae, and lymphocytic infiltration in the superficial layer (4). Palmoplantar keratoderma was observed in both patients in the present series for which topical corticosteroids, emollients, and calcitriol ointment were advised (5).

Immunological abnormalities include low T cells and increased NK cells and immunoglobulins (6). Hyperinflammation leads to defective neutrophil apoptosis, altered nuclear factor-κB signaling, and increased cytokines (7). Both cases in the present series had normal lymphocyte subsets but raised immunoglobulin levels. Deep seated abscesses involving the brain, liver, and kidneys have been reported in patients with PLS (2). Histopathological examination of the abscess has shown chronic granulomatous inflammation around the lesion similar to the histopathology seen in patients with CGD (8). This could be because of the impaired neutrophil function in patients with PLS similar to the neutrophil defect seen in patients with CGD, resulting in inflammatory granuloma (9). In immunocompetent patients, liver abscess usually has a thin rim with central, liquefied, non-enhancing contents, and responds to antibiotic therapy (9). Patients with neutrophil-killing defect and hyperinflammation may have atypical features such as dense, homogeneous with thick inspissated fluid in the liver abscess (9). Index cases had dense organized abscess with calcification. There may be a decreased response to antimicrobials in these cases, and co-administration of corticosteroids may be helpful. Corticosteroids decrease the activation, proliferation, and differentiation of inflammatory cells such as macrophages and lymphocytes (5). Corticosteroids also reduce immune infiltration and capillary leak of central venules, decrease porto-venous shunting, and restore pre-abscess immune milieu (10). This helps in better tissue penetration of antimicrobials because of less-inflamed milieu, particularly surrounding the pseudo-capsule of liver abscess (10). Liver abscess in patients with PLS has been reported to respond to antimicrobials and/or surgical drainage (Supplementary Table 1). In case 1, there was no response despite the surgical drainage and use of antimicrobials. Case 2 did not respond to the antimicrobials. Both cases showed a brisk response to corticosteroids in terms of improvement in fever, inflammatory parameters, and size of the abscess. Corticosteroids may also reduce the chances of recurrence of liver abscess. However, we need more data on these aspects.

## Conclusion

Liver abscess in patients with PLS may need to be treated with corticosteroids along with antimicrobials. This may lead to rapid resolution and possibly reduce the chances of recurrence.

## Data availability statement

The raw data supporting the conclusions of this article will be made available by the authors, without undue reservation.

## Ethics statement

This study was performed in line with the principles of the Declaration of Helsinki. As this manuscript pertains only to case report and literature review, specific ethics approval is not mandated.

## Author contributions

SB, RT, and AZB: data collection, writing the first draft, and editing the manuscript. AKJ: conceptualization, data collection, writing the first draft, editing the manuscript, and final approval of the manuscript. AM, AB, AS, RM, PV, and AR: writing of the initial draft and editing of the manuscript. All authors contributed to the article and approved the submitted version.
