# Supplementary material for: Case report: Corticosteroids as an adjunct treatment for the management of liver abscess in Papillon–Lefèvre syndrome: A report on two cases
Source: Front Pediatr. 2022 Sep 20;10:953033. doi: 10.3389/fped.2022.953033 (PMC9530593; doi:10.3389/fped.2022.953033)
Supplement: Supplementary file 1 [file Table_1.DOCX]

Supplementary Table 1: A review of reported cases of liver abscess in Papillon Lefevre syndrome:

| S. No | Author, year and country | No. of patients | Age  (years) | Sex | Micro-organisms | Treatment |
| --- | --- | --- | --- | --- | --- | --- |
| 1. | Oguzkurt et al, 1996, Turkey(1) | 2 | 8  12 | M  F | *Staphylococcus aureus*  *Escherichia coli* | Antimicrobials, US-guided percutaneous drainage was performed incision and drainage  Antimicrobials, laparotomy, US-guided percutaneous drainage |
| 2. | Khandpur et al, 2001, India(2) | 1 | 14 | M | Not isolated | Antimicrobials , repeated ultrasound guided drainage |
| 3. | Almuneef et al, 2003, Saudi Arabia (3) | 1 | 10 | M | *Staphylococcus aureus* | Antimicrobials , Ultrasound-guided drainage |
| 4. | Dhanawade et al, 2009, India (4) | 1 | 8 | M | Not isolated | Antimicrobials (cefotaxime with sulbactum and amikacin) |
| 5. | Dalgic et al, 2010, Turkey (5) | 1 | 14 | M | *Rhizopus oryzae* | Antimicrobials and antifungal |
| 6. | Mercy et al. 2013 (6) | 1 | 13 | F | Not isolated | Antimicrobials |
| 7. | Das et al, 2013, India (7) | 1 | 15 | F | *Clostridium perfringens, Klebsiella pneumoniae, Enterococcus faecalis* | Antimicrobials |
| 8. | Iqtadar et al, 2015, Pakistan (8) | 1 | 16 | M | *Pseudomonas aeruginosa* | Antimicrobials, Percutaneous drainage |
| 9. | Kartal et al, 2016, Turkey (9) | 1 | 5 | M | *Staphylococcous aureus* | Antimicrobials , surgical drainage |
| 10 | Present study , 2022,India | 2 | 5  6 | M  F | *Staphylococcous aureus*  *Staphylococcous aureus* | Antimicrobials , pig-tail drainage,corticosteroids  Antimicrobials , corticosteroids |

References:

1. Oğuzkurt P,Tanyel FC,Büyükpamukçu N, Hiçsönmez A. Increased risk of pyogenic liver abscess in children with Papillon-Lefevre syndrome.Journal of Pediatric Surgery.1996;31(7):955-956

2. Khandpur S, Reddy BS. Papillon-Lefevre syndrome with pyogenic hepatic abscess: a rare association. Pediatr Dermatol 2001; 18: 45-47

3. Almuneef M, Al Khenaizan S, Al Ajaji S, Al-Anazi A. Pyogenic liver abscess and Papillon-Lefèvre syndrome: not a rare association. Pediatrics. 2003;111(1):e85-88.

4. Dhanawade SS, Shah SD, Kakade GM. Papillon-lefevre syndrome with liver abscess. Indian Pediatr. 2009;46:723–5

5. Dalgic B, Bukulmez A, Sari S. Pyogenic liver abscess and peritonitis due to Rhizopus oryzae in a child with Papillon-Lefevre syndrome. Eur J Pediatr. 2011;170(6):803-5

6.Mercy P, Singh A, Ghorpade AK, Das M, Upadhyay A. Papillon-Lefevre Syndrome: Two Siblings, One Developing Liver Abscess. Indian J Dermatol. 2013;58(5):410.

7.Das SK, Nath T, Ganguly P, Jana CK. A rare case of recurrent pyogenic liver abscess since childhood: A case of Papillon-Lefèvre syndrome. Medical Journal of Dr DY Patil University. 2013;6(1):86.

8. Iqtadar S, Mumtaz SU, Abaidullah S. Papillon-Lèfevre syndrome with palmoplantar keratoderma and periodontitis, a rare cause of pyrexia of unknown origin: a case report. J Med Case Rep. 2015;9:288.

9. Kartal D, Çanar SL, Ferahbaa A, Borlu M, Ukaal U. Papillon Lefevre Syndrome with Hepatic Abscess.Case Report, Clin Dermatol Res J 2016;1(1)
